# Supplementary material for: Incipient diploidization of the medicinal plant Perilla within 10,000 years
Source: Nat Commun. 2021 Sep 17;12:5508. doi: 10.1038/s41467-021-25681-6 (PMC8448860; doi:10.1038/s41467-021-25681-6)
Supplement: Supplementary file 1 — Supplementary Information [file 41467_2021_25681_MOESM1_ESM.docx]

**Incipient diploidization of the medicinal plant *Perilla* within 10,000 years.**

Y. Zhang, Q. Shen, L. Leng, D. Zhang, S. Chen, Y. Shi, Z. Ning, and S. Chen.


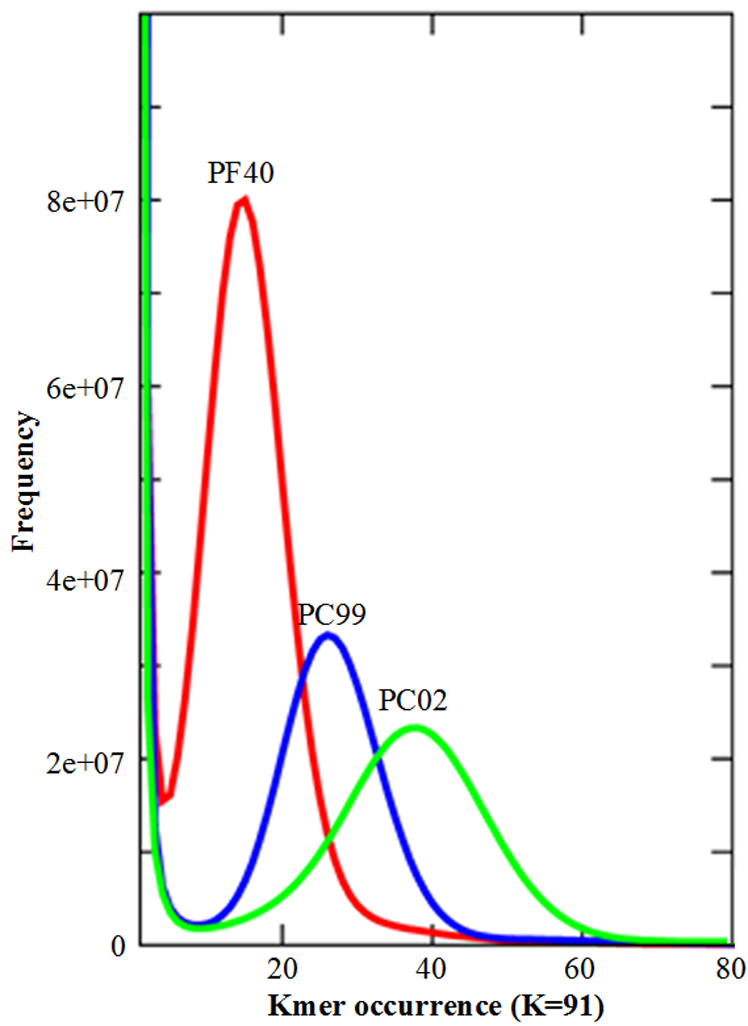


**Supplementary Figure 1. K-mer size estimations of the three perilla genomes.** Kmer occurrence peak values of 17, 26 and 39 were observed for the three genomes. Based on the Kmer frequency data, the genome sizes of PF40, PC02, and PC99 were calculated as 1.24, 0.68, and 0.62 Gb, respectively.


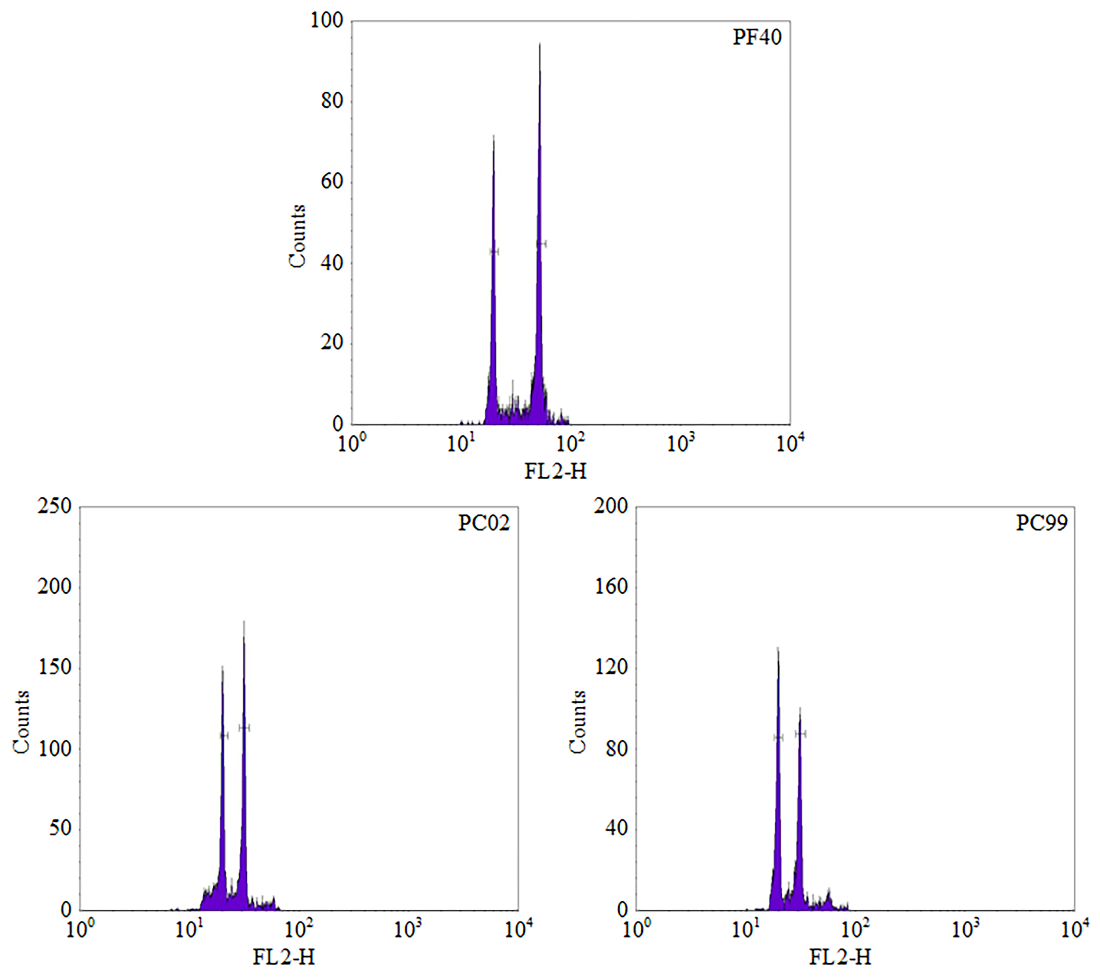


**Supplementary Figure 2. Flow cytometric analysis of the three perilla species.** *Oryza sativa* sp. Japonica Nipponbare (1C = 394.6 Mb, the left peak in each panel) was used as internal reference control for genome size estimation.


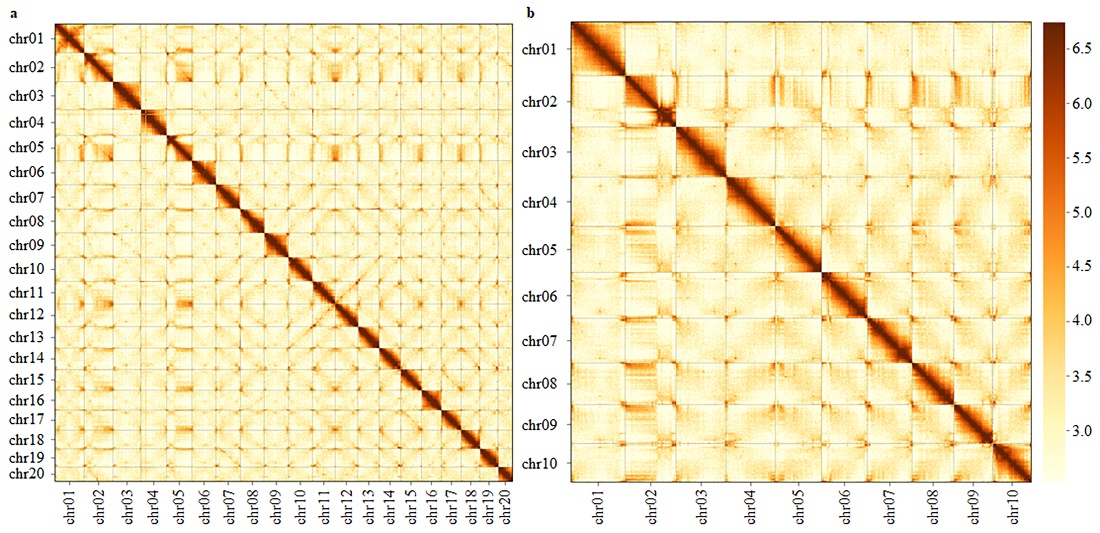


**Supplementary Figure 3. Genome-wide all-against-all Hi-C interaction map of the tetraploid (a) and diploid (b).**


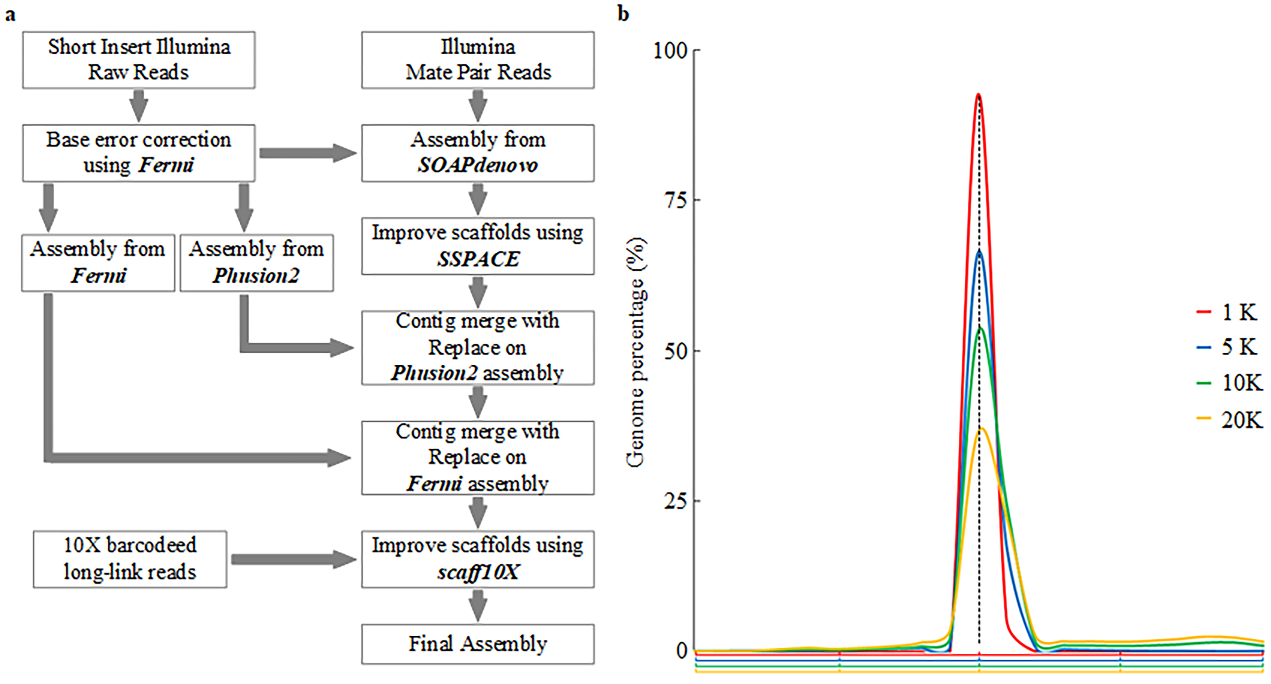


**Supplementary Figure 4. Assembly and evaluation of the Illumina genomes.** (**a**) Flowchart of Illumina pipeline for draft assembly. (**b**) Mapping distance distribution of pseudo mate-pair sequences of Illumina draft assembly on the final PacBio/Hi-C version of PF40. The draft Illumina assembly of PF40 was cut into pseudo mate-pair sequences spanning 1, 5, 10, and 20 Kb, respectively, with read length of 150 bp. These sequences were then mapped onto chromosomal assembly of PF40 by BLASTN, and mapping distance of the top1 hit with correct configuration was kept for statistics. The minimal and maximal values of the X-axis for each size bin were 0.9 and 1.1 kb (red), 4.9 and 5.1 kb (blue), 9 and 11 kb (green), 19 and 21 kb (orange), respectively. It suggested high consistence of the Illumina draft assembly with the PacBio/Hi-C version.


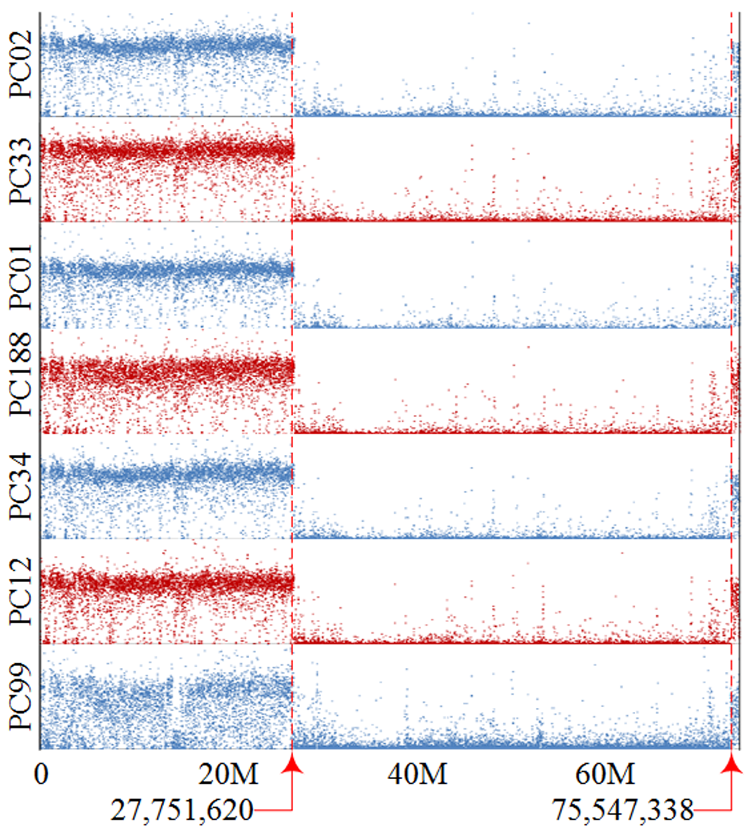


**Supplementary Figure 5. Chimeric coverage depth of seven diploid reads on chr1 of PF40.** The depth was calculated in 5-kb windows. Paucity of coverage around 27.75 – 75.54 Mb interval (Supplementary Table 9) across all samples suggested the same AA-donor attributes of the seven diploids. Average sequencing depth ranged 22 – 33 × for these samples (Supplementary Table 3).


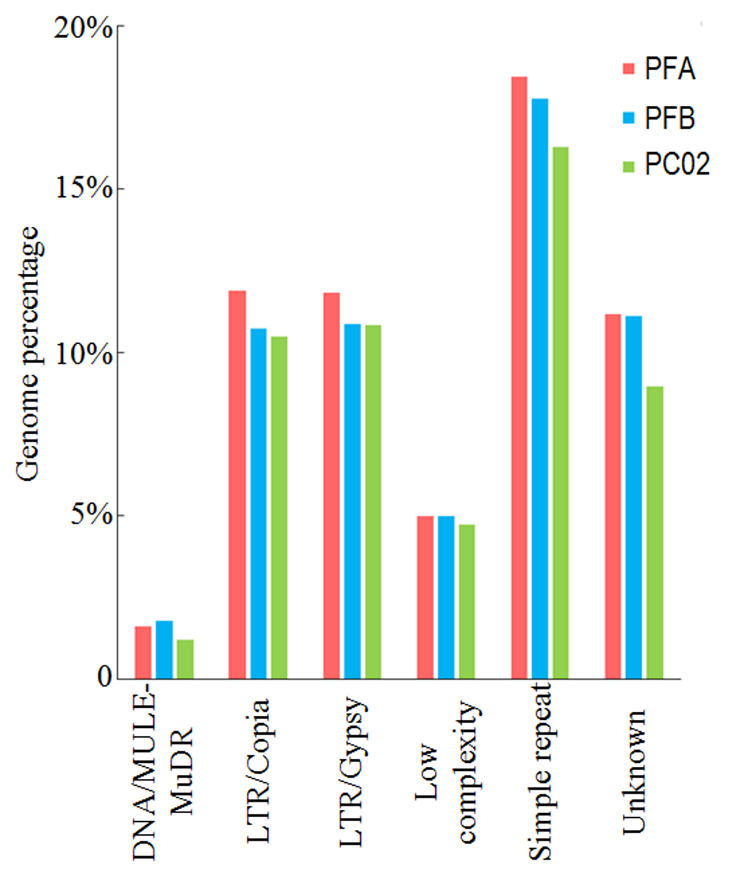


**Supplementary Figure 6. Summary of repeat content of the perilla genomes.** Only the most abundant six types of repeats were shown here. For PFA and PFB data, genome percentage was calculated as that of the PFA and PFB subgenomes, respectively.


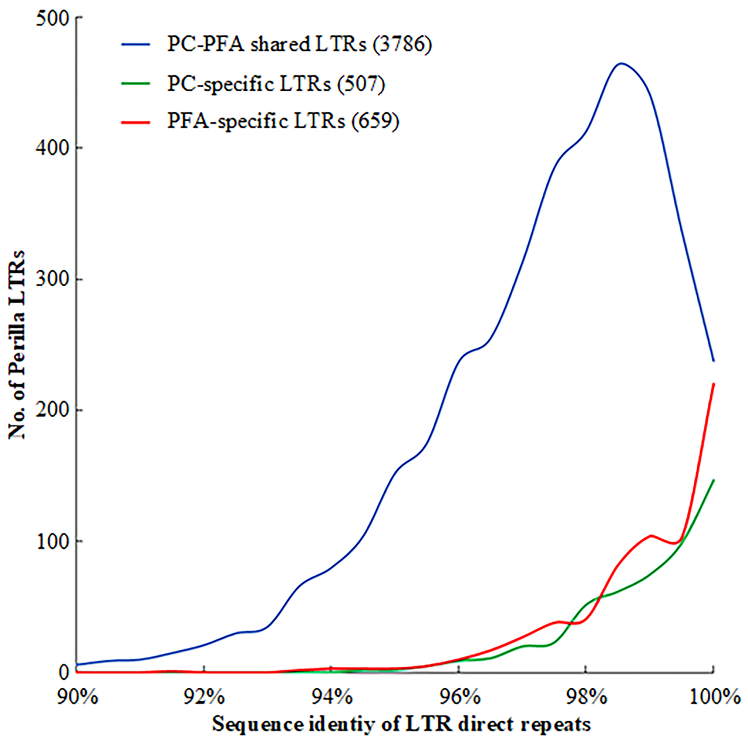


**Supplementary Figure 7. Comparison of LTR-RTs between PFA and PC02.** Totally 5,024 and 5,259 LTR-RTs were identified in PFA and PC02, respectively, and most (3,786) were shared between them. Lineage-specific LTRs, presumably emerged after species divergence, indicated exponential decay of direct repeats of LTRs in both PFA and PC02.


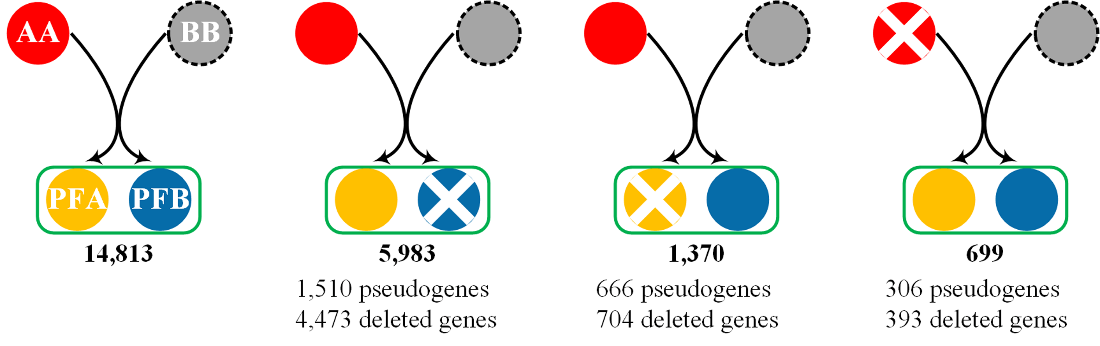


**Supplementary Figure 8. Scenarios and statistics of comparative gene model curations.** Numbers beneath each drawing represent number of genes fit that specific scenario from PFA (orange), PFB (blue), and PC02 (red) sequences. Missing of BB progenitor was shown in grey encircled by dashed line. Overlaid white crosses indicate gene pseudonization or loss within the focal genome. From left to right, genes present in all three genomes, genes not observed in PFB, genes not observed in PFA, and genes not observed in PC02.


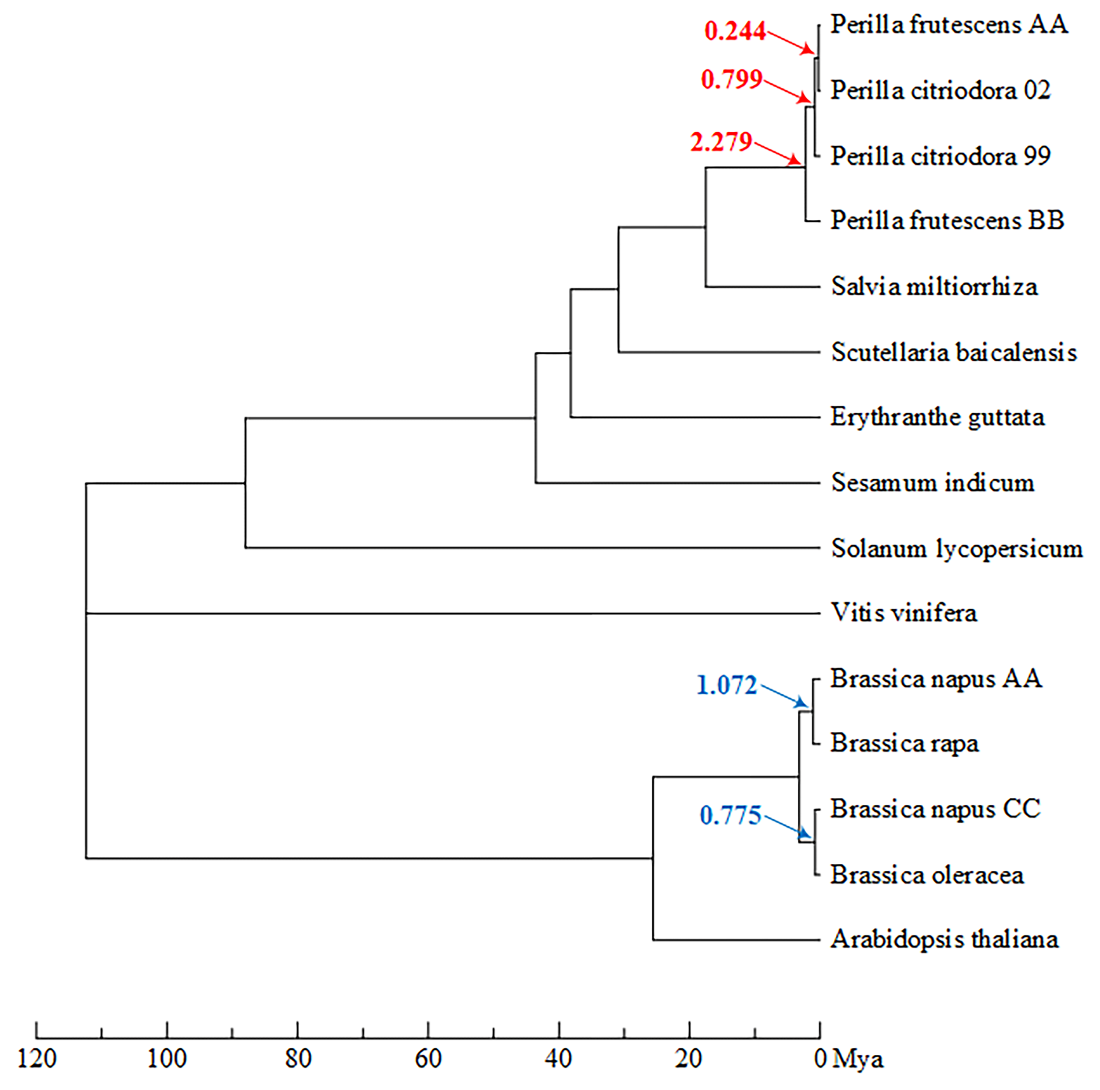


**Supplementary Figure 9.** **Inferred phylogenetic tree across 15 plant genomes.** The tree was based on 606 single copy orthologous genes for 11 plant species and the four perilla genomes.


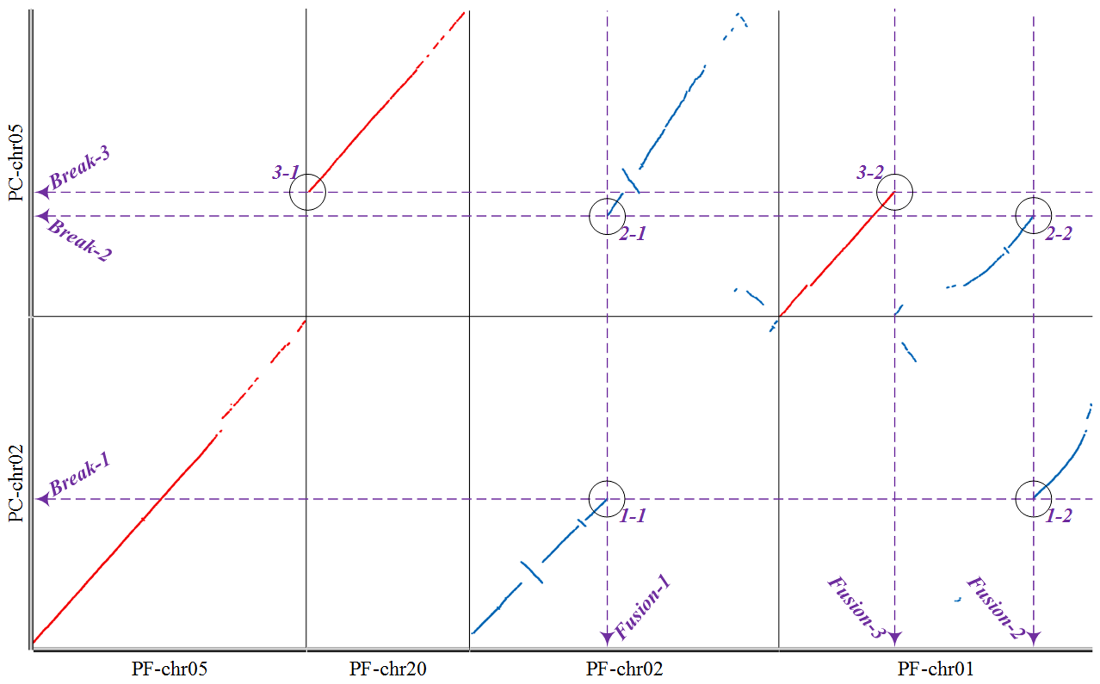


**Supplementary Figure 10. Reshuffling of PF chromosome 1 during diploidization.** Dot plot analysis suggested involvement of three breaks and two fusions for emergence of allotetraploid chr1. Centers of black circles marked breakpoints of the PF chromosomes, and horizontal dashed magenta lines linked two breakpoints from the same breakage event, with arrows pointing to the corresponding PC segments in synteny. Vertical dashed magenta lines indicated three fusions specific to PF. Break-3 of the AA subgenome split the 1-aminocyclopropane-1-carboxylate synthase gene (ACS) in the second intron. ACS is a direct precursor of ethylene, so truncation of the AA-derived ACS gene might compromise ethylene synthesis and signaling of the tetraploid.


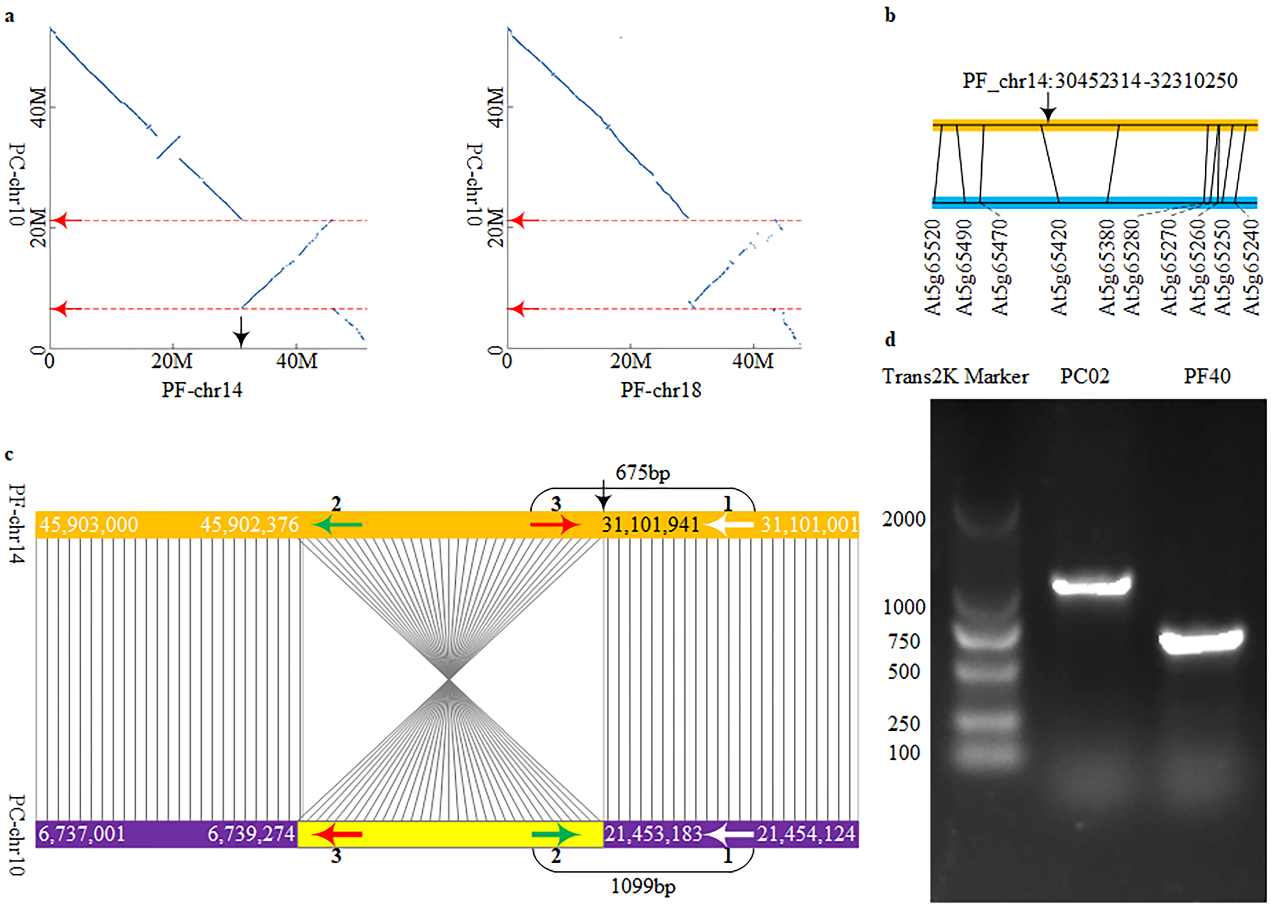


**Supplementary Figure 11. Identification of a PC-specific inversion of 14.8 Mb.** (**a**) Dot plot analysis indicated shared 14.8 Mb inversions of PF chr14 and chr18 relative to PC chr10. Dashed red lines pointed to breakpoint correspondence on PC chromosome, and black arrowhead indicated potential breakpoint of PF chr14 that was further analyzed in (**b**) to (**d**). (**b**) Interval of PF chr14 around the upper breakpoint indicated micro-synteny with Arabidopsis chr5, suggesting that PF represented intact ancestral karyotype. Arrowhead pointed to the same position as in (**a**). (**c**) Schematic representation of homologous relationship around inversion breakpoints. Colored arrowheads represented primer positions for PCR validation. (**d**) Gel electrophoresis of PCR products with primer 1 + 2 + 3 (Supplementary Table 14). Trans2K DNA Marker (TransGene, Beijing) was used, with band size marked to the left. Only the 1 + 2 band was amplified in PC02 (1,099 bp), and only the 1 + 3 band was amplified in PF40 (675 bp), implied correctness of both assemblies.


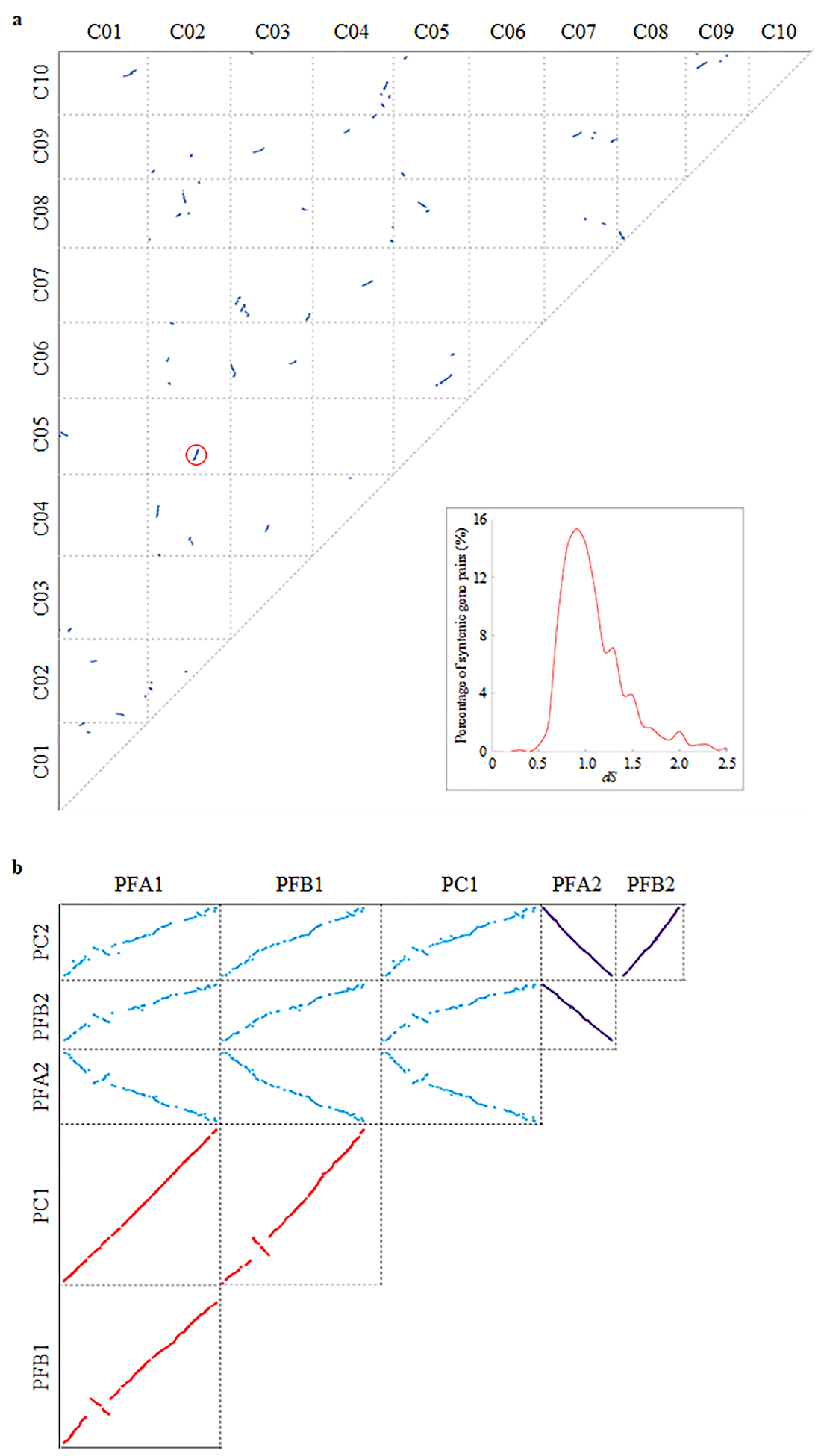


**Supplementary Figure 12. Genome synteny of the diploid PC02 genome.** (**a**) Dot-plot analysis of PC02 genes against PC02 to identify signatures of ancient polyploidy. Each dot represented syntenic gene relationship within PC02 genome. Totally 1,812 duplicated gene pairs were identified (at least 8 anchor gene pairs required for each syntenic block). The *dS* distribution plot was shown as inset. Red circle highlighted a WGD signal between chr02 and chr05 of PC02, which were further analyzed across diploid and tetraploid in (**b**). (**b**) Ancient WGD signals were also observed within PFA and PFB subgenomes. Coordinates for these six segments were chr01:12.686-22.484M (PFA1), chr01:52.084-61.162M (PFB1), chr05:11.735-21.407M (PC1), chr05:23.938-28.395M (PFA2), chr01:68.327-71.979M (PFB2), and chr02:38.525-42.918M (PC2).


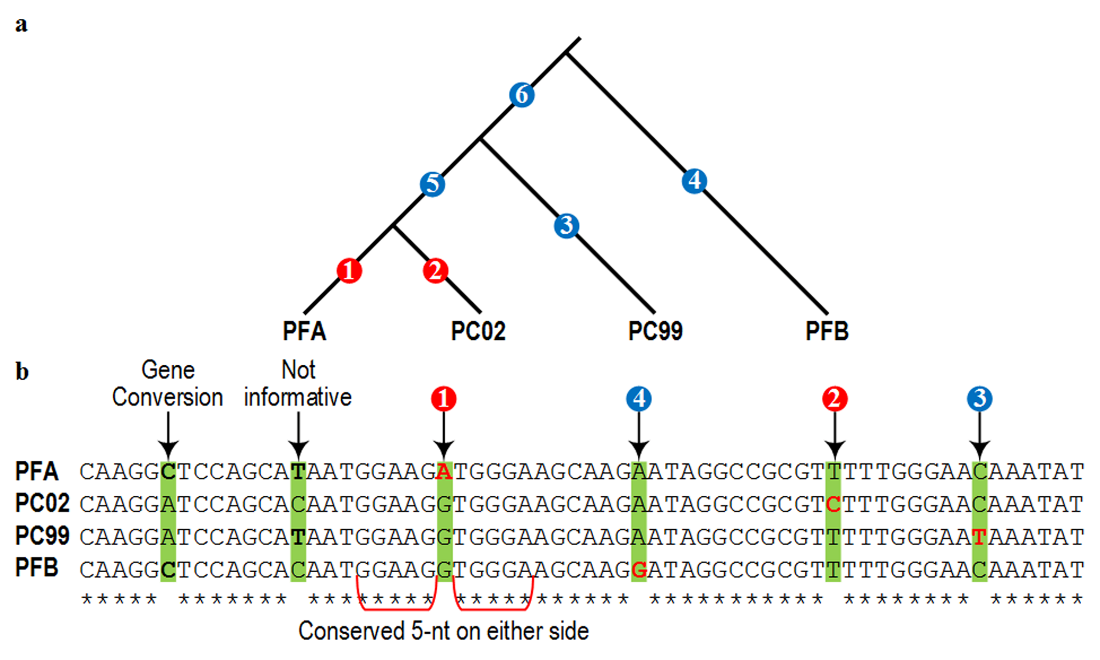


**Supplementary Figure 13. Rationales of nucleotide mutation identification.** (**a**) Simplified NJ-tree of the four sequences. Six scenarios of mutation occurrence were labelled along the branches, among which only PFA (scenario 1) or PC02 (scenario 2) specific mutations were included for further analysis. (**b**) For each MAFFT alignment interval, only those 1:3 genotype-count sites were informative as one mutation (marked with red letter) and three ancestral nucleotides, and each confirmed mutation must be flanked by 5 consecutive nucleotides on either side that are identical across the four sequences, to rule out false positive identifications from ambiguous local alignment. Gene conversions between PFA and PFB will introduce abnormal tree topology and different genotype-count (4:0 or 2:2). These substitutions were not resulted from *de novo* mutations, and were excluded in our analysis. Note that scenario 4 is equivalent to scenario 6, and cannot be determined definitely without outgroup information.


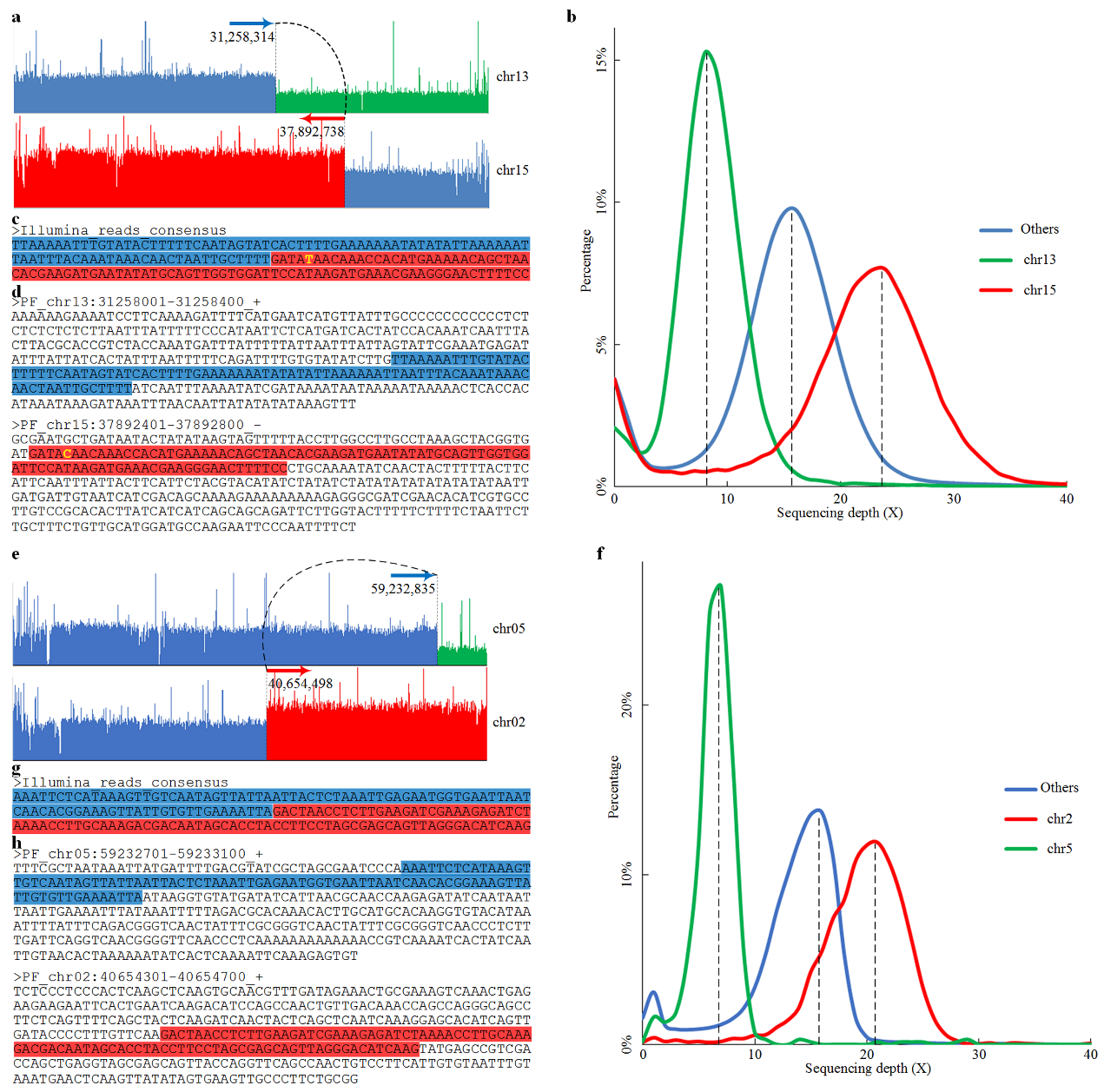


**Supplementary Figure 14. Genome aneuploidy of the allotetraploid from non-homeologous aberrant exchanges.** (**a** – **d**) Aneuploidy in accession PF175. (**a**) Sequencing depth of the two affected chromosomes. Depth data were calculated in 5-kb windows. Blue, green, and red lines indicated regions with genome average depth (2 ×), half depth (1 ×, deletion), and 3 × depth (duplication), respectively. (**b**) Depth distribution plot of PF175. “Others” data was from the remaining normal segments of the genome. (**c**) Illumina consensus sequence by local assembly of reads spanning the exchange breakpoint (dashed line that linked blue and red arrowheads in **a**). Color code was the same as in (**a**) and (**b**). (**d**) Sequences of the reference PF genome around the breakpoints. It suggested that 25.25 Mb sequences of chr13 was completely replaced by 37.89 Mb from chr15 in PF175. One C/T mutation was introduced by this rearrangement as highlighted in yellow. (**e** – **h**) Aneuploidy from aberrant exchange between chr2 and chr5 in PF007.


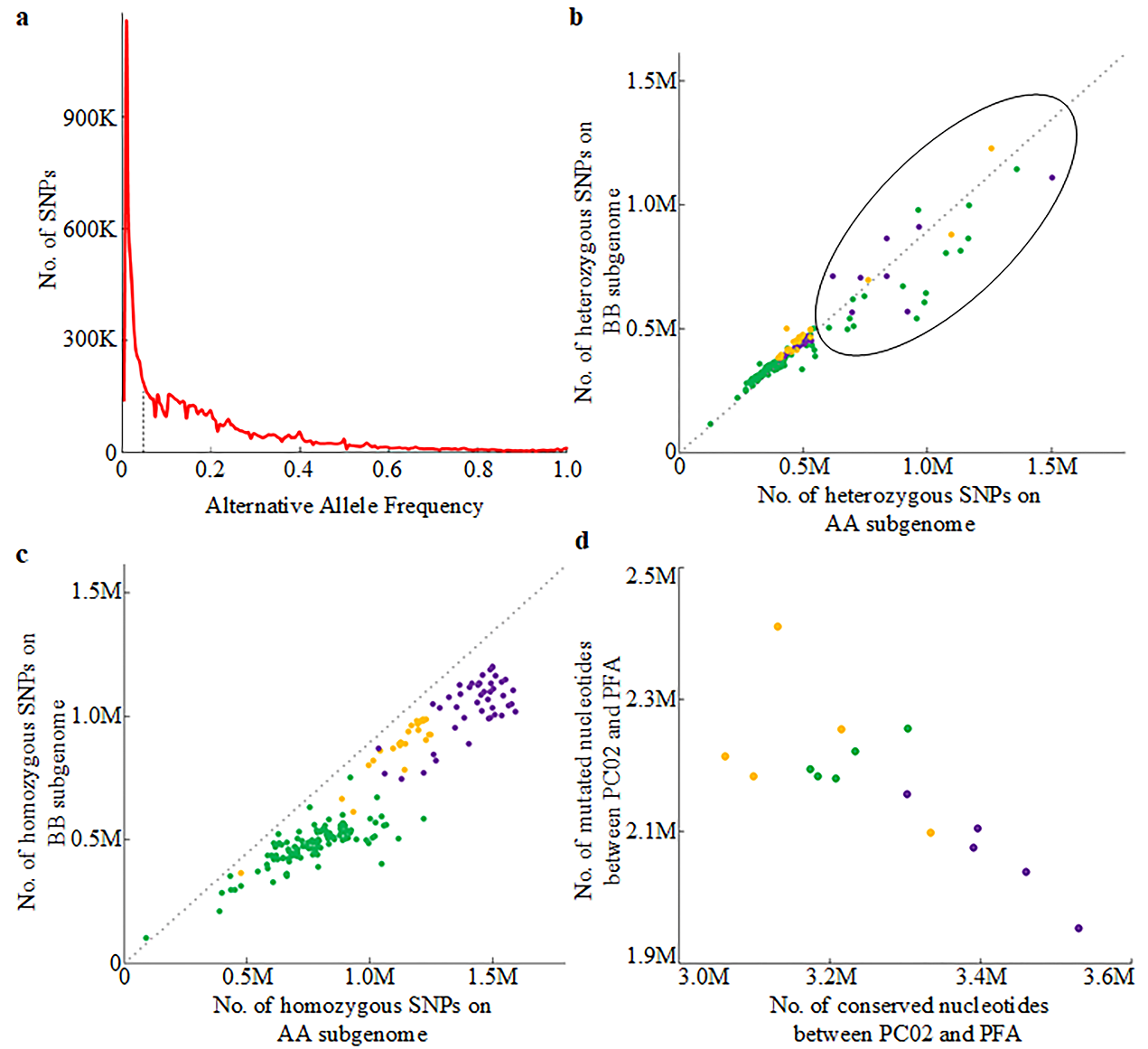


**Supplementary Figure 15. Genomic polymorphism of the resequencing population.** (**a**) Distribution of alternative allele frequencies of SNPs of the 191 tetraploid accessions. PF40 is taken as the proto-genotype, and dashed grey lines represented values of 0.05. (**b**) Distribution of heterozygous SNPs between PFA and PFB from each sample. Dashed grey line represented the same SNP density on PFA and PFB. Color code of the 191 samples was the same as in Fig.4. Low level of heterozygotes that evenly distributed on PFA and PFB corroborated the selfing nature of perilla, and outliers encircled by oval implied genetic admixture possibly from interbreeding. (**c**) Distribution of homozygous SNPs between PFA and PFB. (**d**) Patterns of polymorphism of 15 tetraploid lines (five from each clade) against the diploid PC02 genome. Totally 6,599,943 SNPs not completely fixed in tetraploid yet were counted for each line.


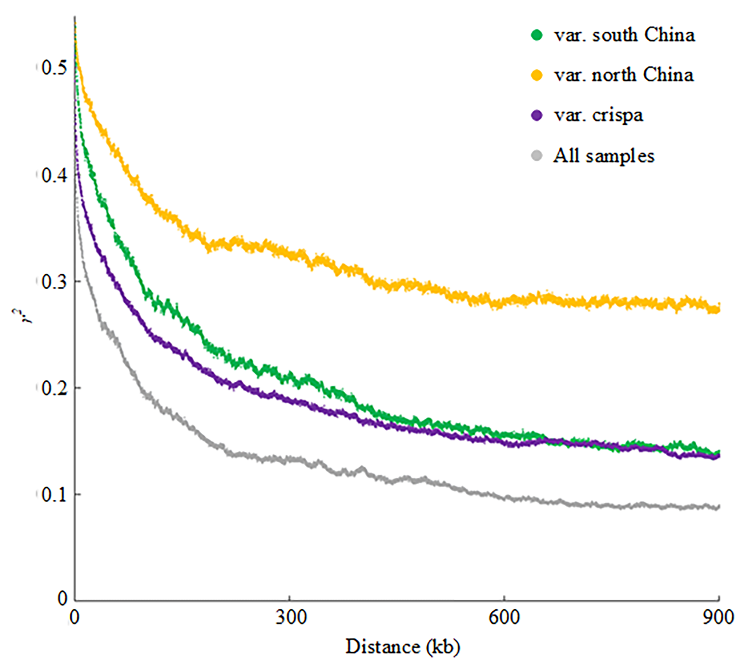


**Supplementary Figure 16. Plot of genome-wide LD decay for different perilla clades.**


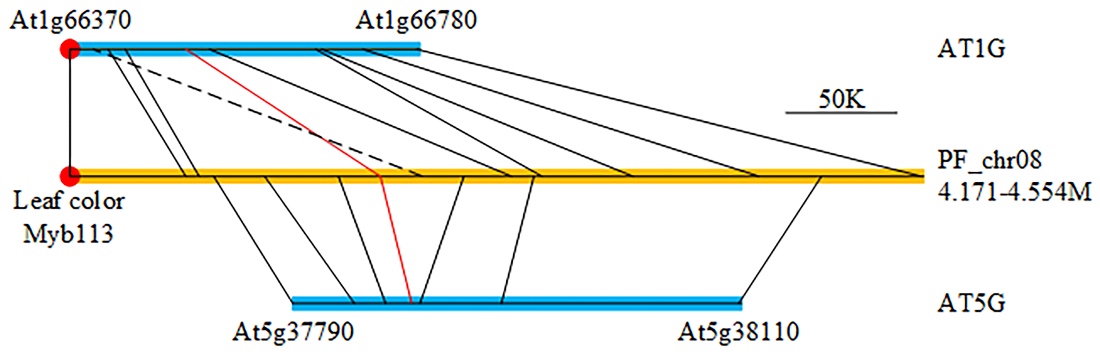


**Supplementary Figure 17. Micro-synteny of Myb113 loci between Perilla and Arabidopsis.** Solid lines represented syntenic gene pairs between two chromosomes, and dashed line indicated syntenic genes with local rearrangements. There were 10 and 7 anchor genes on chr1 and chr5 of Arabidopsis, respectively, that had syntenic relationship with perilla, but only one syntenic gene pair can be inferred between the two Arabidopsis segments (At1g66480 *vs.* At5g37840, linked with red line). Such “ghost duplication”^1^ suggested that differential gene loss of two originally identical duplicated regions extensively blurred syntenic relationship within a single genome, while comparative genomics can recover hidden block duplications that had seemingly disappeared by introducing a third genome.


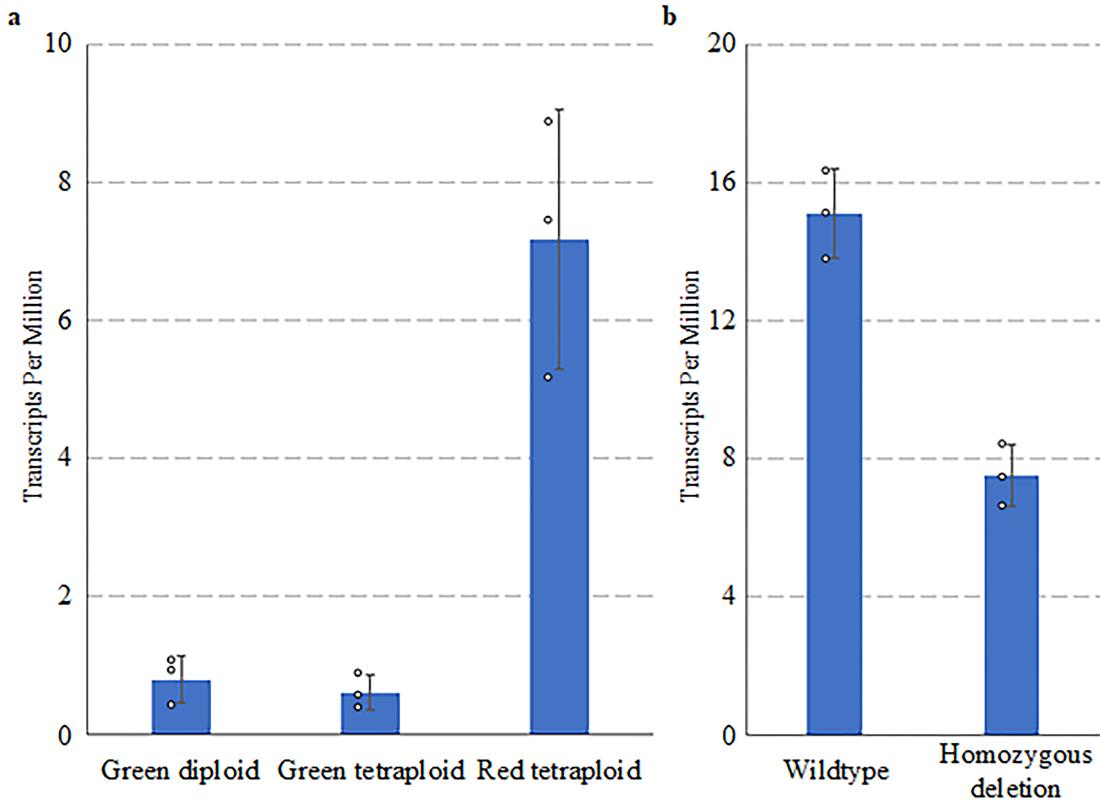


**Supplementary Figure 18. Expression of candidate genes across different genotypes of perilla.** (**a**) TPM values of the leaf-color Myb113 gene in perilla. “Green diploid” was from three replicates of RNAseq with PC02 green leaves, “Green tetraploid” was extracted from RNAseq of PF40, PF100, and PF240 (all with mutated Myb113 and no upstream deletion), and “Red tetraploid” was extracted from PF008, PF010, and PF183 ((all with intact Myb113 and upstream deletion). The analyzed Myb113 gene was chr8:4171570-4174541 in PF40 and chr4:64042835-64045824 in PC02, respectively. Error bars, mean ± s.d. (**b**) TPM values of LPCAT gene in tetraploid perilla. “Wildtype” was extracted from flower RNAseq of PF016, PF021, and PF033, and “Homozygous deletion” was from flower RNAseq of PF084, PF089, and PF098.

**Supplementary Table 1. Perilla genome assembly statistics.**

|  | PF40 | | PC02 | |
| --- | --- | --- | --- | --- |
|  | Contig | Pseudo-molecule | Contig | Pseudo-molecule |
| Total length | 1,241,495,061 | 1,234,377,696 | 680,845,938 | 676,955,838 |
| No. sequences | 2,098 | 1,467 | 2,824 | 1,791 |
| Length N50 | 3,211,575 | 62,644,896 | 955,603 | 64,469,250 |
| L50 | 113 | 10 | 202 | 5 |
| Length N80 | 1,441,366 | 52,707,976 | 401,270 | 54,042,029 |
| L80 | 282 | 16 | 536 | 9 |
| Longest length | 17,112,643 | 76,507,337 | 5,600,750 | 75,432,221 |

**Supplementary Table 2. Statistics of chromosome, annotation, and synteny of PF40 genome.**

| PF40 | Length (bp) | No. of Scaffolds | No. of Annotated Genes | No. of Anchor genes on PFA | No. of Anchor Genes on PFB |
| --- | --- | --- | --- | --- | --- |
| chr01 | 76,507,337 | 31 | 3,246 | 1,245 | 1,615 |
| chr02 | 75,925,849 | 45 | 3,625 | 0 | 3,095 |
| chr03 | 73,186,951 | 63 | 1,837 | 1,501 | 0 |
| chr04 | 68,127,393 | 49 | 2,106 | 1,821 | 0 |
| chr05 | 66,039,351 | 40 | 3,648 | 3,337 | 0 |
| chr06 | 63,979,823 | 44 | 1,825 | 1,464 | 0 |
| chr07 | 64,111,010 | 39 | 1,961 | 1,592 | 35 |
| chr08 | 62,644,896 | 46 | 2,012 | 1,735 | 0 |
| chr09 | 63,810,348 | 28 | 1,555 | 0 | 1,228 |
| chr10 | 63,122,535 | 37 | 2,133 | 1,841 | 0 |
| chr11 | 59,720,668 | 51 | 2,565 | 227 | 1,953 |
| chr12 | 59,655,398 | 49 | 2,690 | 2,407 | 0 |
| chr13 | 56,513,981 | 33 | 1,779 | 0 | 1,461 |
| chr14 | 56,187,003 | 57 | 1,940 | 1,703 | 0 |
| chr15 | 54,298,802 | 23 | 2,236 | 2,025 | 0 |
| chr16 | 52,707,976 | 35 | 1,614 | 0 | 1,336 |
| chr17 | 52,085,854 | 29 | 1,970 | 0 | 1,713 |
| chr18 | 49,030,717 | 29 | 1,667 | 0 | 1,399 |
| chr19 | 47,713,458 | 30 | 1,340 | 0 | 1,113 |
| chr20 | 37,888,901 | 25 | 1,283 | 1,140 | 10 |
| un-anchored | 31,119,445 | 1,447 | 495 | - | - |

**Supplementary Table 3. Evaluation of seven diploid samples by Illumina reads mapping on PF genome.**

| Samples | Habitat | Latitude and longitude | Clean data (Gb) | Mapping % on PF40 | Coverage % of PF40 | Depth (X) |
| --- | --- | --- | --- | --- | --- | --- |
| PC02 | Tianmu Mountain Nature Reserve, Zhejiang Province | 30°21'N, 119°26'E | 20.07 | 99.37% | 57.38% | 27 |
| PC33 | Tianmu Mountain Nature Reserve, Zhejiang Province | 30°21'N, 119°26'E | 19.59 | 99.12% | 57.27% | 27 |
| PC01 | Tianmu Mountain Nature Reserve, Zhejiang Province | 30°21'N, 119°26'E | 16.71 | 99.03% | 56.82% | 22 |
| PC188 | Maolan Karst Forest, Guizhou Province | 25°11'N, 108°12'E | 23.85 | 98.93% | 60.18% | 33 |
| PC34 | Tianmu Mountain Nature Reserve, Zhejiang Province | 30°21'N, 119°26'E | 19.96 | 96.85% | 57.45% | 25 |
| PC12 | Maolan Karst Forest, Guizhou Province | 25°11'N, 108°12'E | 17.49 | 96.68% | 57.64% | 24 |
| PC99 | Tianmu Mountain Nature Reserve, Zhejiang Province | 30°21'N, 119°26'E | 18.02 | 92.27% | 59.24% | 23 |

**Supplementary Table 4. Statistics of chromosome, annotation, and synteny of PC02 genome.**

| PC02 | Length (bp) | No. of Scaffolds | No. of Annotated Genes | No. of Anchor Genes with PF |
| --- | --- | --- | --- | --- |
| chr01 | 75,432,221 | 164 | 1,941 | 1,523 |
| chr02 | 70,609,245 | 103 | 4,049 | 3,565 |
| chr03 | 69,736,025 | 139 | 2,303 | 1,979 |
| chr04 | 68,219,680 | 131 | 2,338 | 1,781 |
| chr05 | 64,469,250 | 103 | 2,707 | 2,379 |
| chr06 | 63,953,966 | 100 | 2,101 | 1,649 |
| chr07 | 62,397,036 | 92 | 2,178 | 1,873 |
| chr08 | 57,865,665 | 100 | 2,741 | 2,469 |
| chr09 | 54,042,029 | 87 | 2,357 | 2,108 |
| chr10 | 53,036,168 | 90 | 2,001 | 1,747 |
| un-anchored | 37,194,553 | 1,781 | 946 | - |

**Supplementary Table 5. Assembly statistics of the three perilla genomes by Illumina procedure.**

| PF40 |  | Contig | | Scaffold | |
| --- | --- | --- | --- | --- | --- |
|  |  | Size (bp) | Number | Size (bp) | Number |
|  | N50 | 92,392 | 3,954 | 838,934 | 438 |
|  | N60 | 73,096 | 5,461 | 652,386 | 607 |
|  | N70 | 54,508 | 7,418 | 484,920 | 832 |
|  | N80 | 36,684 | 10,173 | 320,058 | 1,156 |
|  | N90 | 17,293 | 14,913 | 140,825 | 1,725 |
|  | Longest | 879,910 | - | 4,541,986 | - |
|  | Total Size | 1,238,411,774 | 78,897 | 1,254,903,076 | 25,684 |
| PC02 | N50 | 68,758 | 2,843 | 1,168,461 | 172 |
|  | N60 | 54,321 | 3,911 | 931,321 | 236 |
|  | N70 | 41,324 | 5,289 | 697,215 | 318 |
|  | N80 | 28,185 | 7,188 | 499,401 | 431 |
|  | N90 | 13,448 | 10,437 | 273,021 | 606 |
|  | Longest | 537,248 | - | 5,524,780 | - |
|  | Total Size | 653,691,640 | 47,916 | 673,748,395 | 25,080 |
| PC99 | N50 | 79,801 | 2,285 | 477,949 | 371 |
|  | N60 | 62,209 | 3,153 | 373,009 | 518 |
|  | N70 | 47,895 | 4,270 | 278,555 | 709 |
|  | N80 | 33,101 | 5,789 | 163,761 | 996 |
|  | N90 | 15,398 | 8,374 | 55,694 | 1,604 |
|  | Longest | 561,772 | - | 5,063,236 | - |
|  | Total Size | 610,931,429 | 47,371 | 618,811,097 | 32,473 |

**Supplementary Table 6. Summary of Illumina sequencing data for draft assembly and evaluation.**

| Sample | Insert size | Read length (bp) | Clean data (Gb) | Mapping % | PCR duplication % | Coverage % | Depth (X) |
| --- | --- | --- | --- | --- | --- | --- | --- |
| PF40 | 500bp | 150 | 33.66 | 98.99% | 14.63% | 98.54% | 20 |
|  | 800bp | 250 | 93.59 | 98.11% | 5.52% | 97.16% | 71 |
|  | 2Kb | 125 | 16.09 | 99.79% | 2.45% | 97.13% | 10 |
|  | 5Kb | 125 | 31.37 | 99.84% | 9.62% | 97.50% | 19 |
| PC02 | 500bp | 150 | 66.38 | 99.05% | 10.19% | 96.69% | 99 |
|  | 800bp | 250 | 57.92 | 98.75% | 6.87% | 95.59% | 108 |
|  | 2Kb | 150 | 8.93 | 96.44% | 15.88% | 96.49% | 11 |
|  | 5Kb | 150 | 8.49 | 95.51% | 66.38% | 91.92% | 8 |
|  | 10Kb | 150 | 5.61 | 91.80% | 41.70% | 94.07% | 6 |
| PC99 | 500bp | 150 | 50.29 | 98.46% | 12.07% | 98.42% | 71 |
|  | 800bp | 250 | 59.08 | 96.18% | 4.77% | 94.22% | 125 |
|  | 5Kb | 150 | 5.26 | 94.93% | 68.39% | 81.64% | 3 |

**Supplementary Table 7. Evaluation of PF40 assembly by published EST and RNAseq data.**

| Source | ^§^EST | Green leaf RNAseq (ref. 17) | Red leaf RNAseq (ref. 17) | RNAseq (ref. 12) |
| --- | --- | --- | --- | --- |
| No. Sequences | 5,538 | 54,445 | 54,500 | 54,079 |
| Total Length (bp) | 4,297,859 | 45,949,783 | 44,923,850 | 47,146,440 |
| *Mapped No. | 5,350 | 53,043 | 53,463 | 53,151 |
| Mapped Length (bp) | 4,137,794 | 44,271,365 | 43,420,022 | 46,073,214 |
| Mapping percentage | 96.28% | 96.35% | 96.65% | 97.72% |

*Blast identity≥95%

^§^Perilla EST downloaded from GenBank as of 2019-10-01

**Supplementary Table 8. BUSCO evaluation of the three perilla genomes.**

| Evaluation of genome assembly | Category | PF40 | PC02 | PC99 |
| --- | --- | --- | --- | --- |
|  | Complete BUSCOs | 1,335 | 1,333 | 1,326 |
|  | Complete and single-copy BUSCOs | 473 | 1,249 | 1,241 |
|  | Complete and duplicated BUSCOs | 862 | 84 | 85 |
|  | Fragmented BUSCOs | 18 | 23 | 23 |
|  | Missing BUSCOs | 87 | 84 | 91 |
|  | Total BUSCO groups searched | 1,440 | 1,440 | 1,440 |
|  | Percentage of Complete | 92.71% | 92.57% | 92.08% |
| Evaluation of gene annotation | Complete BUSCOs | 1,292 | 1,260 | 1,239 |
|  | Complete and single-copy BUSCOs | 274 | 1,183 | 1,169 |
|  | Complete and duplicated BUSCOs | 1,018 | 77 | 70 |
|  | Fragmented BUSCOs | 32 | 32 | 33 |
|  | Missing BUSCOs* | 116 | 148 | 168 |
|  | Total BUSCO groups searched | 1,440 | 1,440 | 1,440 |
|  | Percentage of Complete | 89.70% | 87.50% | 86.10% |
| Retrieved pseudogene annotations | by polymorphic SNPs | 5 | 4 | 6 |
|  | by polymorphic Indels | 20 | 28 | 18 |
|  | by fixed SNPs | 5 | 13 | 9 |
|  | by fixed Indels | 44 | 60 | 49 |

*Those "missing BUSCOs" were firstly retrieved from embryophyta_odb9 database, then searched against the three perilla genomes, to evaluate reasons of their omission.

**Supplementary Table 9. Subgenome assignment of PF40.**

| PF_chr | From | To | Origin |
| --- | --- | --- | --- |
| chr01 | 1 | 27,751,620 | AA |
| chr01 | 27,751,621 | 75,547,337 | BB |
| chr01 | 75,547,338 | 76,507,337 | AA |
| chr02 | 1 | 75,925,849 | BB |
| chr03 | 1 | 73,186,951 | AA |
| chr04 | 1 | 68,127,393 | AA |
| chr05 | 1 | 66,039,351 | AA |
| chr06 | 1 | 63,979,823 | BB |
| chr07 | 1 | 62,940,971 | AA |
| chr07 | 62,940,972 | 64,111,010 | BB |
| chr08 | 1 | 62,644,896 | AA |
| chr09 | 1 | 63,810,348 | BB |
| chr10 | 1 | 63,122,535 | AA |
| chr11 | 1 | 2,396,516 | AA |
| chr11 | 2,396,517 | 59,720,668 | BB |
| chr12 | 1 | 59,655,398 | AA |
| chr13 | 1 | 56,513,981 | BB |
| chr14 | 1 | 56,187,003 | AA |
| chr15 | 1 | 54,298,802 | AA |
| chr16 | 1 | 52,707,976 | BB |
| chr17 | 1 | 52,085,854 | BB |
| chr18 | 1 | 49,030,717 | BB |
| chr19 | 1 | 47,713,458 | BB |
| chr20 | 1 | 37,302,395 | AA |
| chr20 | 37,302,396 | 37,888,901 | BB |

Note: All of the five PFA-PFB junctions were validated by PCRs.

**Supplementary Table 10. Statistics of tRNAs of the perilla genomes.**

|  | PF40 | | PC02 | |
| --- | --- | --- | --- | --- |
|  | Raw | Filtered | Raw | Filtered |
| tRNAs decoding Standard 20 AA | 1,316 | 1,162 | 793 | 669 |
| Selenocysteine tRNAs (TCA) | 1 | 0 | 0 | 0 |
| Possible suppressor tRNAs (CTA,TTA) | 3 | 1 | 0 | 0 |
| tRNAs with undetermined/unknown isotypes | 3 | 2 | 6 | 1 |
| Predicted pseudogenes | 146 | 0 | 75 | 0 |
| Sum | 1,469 | 1,165 | 874 | 670 |

**Supplementary Table 11. Statistics of other non-coding RNAs identified in the perilla genomes.**

|  | PF40 | | | PC02 | | |
| --- | --- | --- | --- | --- | --- | --- |
|  | No. | Sum | Average | No. | Sum | Average |
| rRNA | 1,697 | 468,687 | 276.2 | 709 | 225,953 | 318.7 |
| snoRNA | 69 | 5,358 | 77.7 | 35 | 2,759 | 78.8 |
| microRNA | 210 | 27,610 | 131.5 | 108 | 14,087 | 130.4 |
| snRNA | 71 | 15,089 | 212.5 | 10 | 2,169 | 216.9 |
| snoRNA | 157 | 15,108 | 96.2 | 82 | 7,904 | 96.4 |

**Supplementary Table 12. Summary of gene family information.**

| Species | No. predicted genes^1^ | No. genes used^2,3^ | No. genes clustered | % |
| --- | --- | --- | --- | --- |
| *Arabidopsis thaliana* | 48,265 | 25,113 | 23,559 | 93.8 |
| *Brassica napus AA* | 123,467 | 45,963 | 44,204 | 96.2 |
| *Brassica napus CC* |  | 47,045 | 45,466 | 96.6 |
| *Brassica oleracea* | 56,687 | 42,816 | 41,628 | 97.2 |
| *Brassica rapa* | 60,607 | 39,886 | 39,150 | 98.2 |
| *Mimulus guttatus* | 31,861 | 26,659 | 24,687 | 92.6 |
| *Perilla citriodora 02* | 25,662 | 23,973 | 23,605 | 98.5 |
| *Perilla citriodora 99* | 23,819 | 22,430 | 22,035 | 98.2 |
| *Perilla frutescens AA* | 23,549 | 22,023 | 21,625 | 98.2 |
| *Perilla frutescens BB* | 19,978 | 19,041 | 18,593 | 97.6 |
| *Scutellaria baicalensis* | 30,313 | 27,363 | 24,494 | 89.5 |
| *Sesamum indicum* | 35,410 | 23,431 | 22,495 | 96.0 |
| *Solanum lycopersicum* | 37,660 | 24,973 | 23,023 | 92.2 |
| *Salvia miltiorrhiza* | 29,236 | 27,658 | 24,960 | 90.2 |
| *Vitis vinifera* | 41,208 | 25,104 | 22,622 | 90.1 |

Note:

1. Total predicted genes in respective genome release;

2. For multiple isoform predictions within each genome, only the prediction with longest protein sequence was kept for analysis;

3. Predicted proteins shorter than 100aa were filtered out;

**Supplementary Table 13. Statistics of inter-specific variations by mapping PC02 onto PF genome.**

|  | Total length (bp) | No. of SNPs | No. of Indels | SNP density (/kb) |
| --- | --- | --- | --- | --- |
| Exonic sequences | 32,719,716 | 62,192 | 12,026 | 1.90 |
| Intronic sequences | 44,041,265 | 110,480 | 50,977 | 2.51 |
| Intergenic regions | 557,852,850 | 2,266,568 | 586,285 | 4.06 |

**Supplementary Table 14. PCR primers for validation of the 14.8 Mb inversion of PC02.**

| Oligos | Coordinate in PF40 | Coordinate in PC02 | Sequences |
| --- | --- | --- | --- |
| 1 | chr14:31101645-31101664 | chr10:21453461_21453480 | GAACACCCGCTATCCAGGTA |
| 2 | chr14:45901574_45901593 | chr10:21452382_21452401 | TCCGGCAACTTAGTTTCGAT |
| 3 | chr14:31102300_31102319 | chr10:06739545_06739564 | GCCCCACTCAACAGAATCAT |

**Supplementary Table 15. Statistics of identified nucleotide mutations across four subgenomes.**

|  | Transitions | | Transversions | | | | Total | Ts/Tv |
| --- | --- | --- | --- | --- | --- | --- | --- | --- |
|  | A:T→G:C | C:G→T:A | A:T→C:G | A:T→T:A | C:G→A:T | C:G→G:C |  |  |
| PFA | 50,842 (24.67%) | 82,290 (39.93%) | 14,517 (7.04%) | 24,572 (11.92%) | 21,455 (10.41%) | 12,393 (6.01%) | 206,069 | 1.83 |
| PC02 | 47,033 (25.13%) | 74,779 (39.95%) | 13,132 (7.02%) | 22,242 (11.88%) | 18,892 (10.09%) | 11,103 (5.93%) | 187,181 | 1.86 |
| PC99 | 175,246 (29.27%) | 209,688 (35.03%) | 43,586 (7.28%) | 75,169 (12.56%) | 60,250 (10.06%) | 34,723 (5.80%) | 598,662 | 1.80 |
| PFB | 1,091,500 (29.68%) | 1,161,926 (31.59%) | 326,622 (8.88%) | 517,714 (14.08%) | 339,499 (9.23%) | 240,634 (6.54%) | 3,677,895 | 1.58 |
| PFA abundance over PC02 | 8.1% | 10.0% | 10.5% | 10.5% | 13.6% | 11.6% | 10.1% | - |

**Supplementary Table 16. List of 18 balanced HEs as reciprocal swap of syntenic PFA and PFB segments.**

| PFB-sequences within AA block | PFA-sequences within BB block |
| --- | --- |
| chr03:13099000-13112000 | chr09:09970000-10007500 |
| chr03:72153500-72167000 | chr09:60154500-60203000 |
| chr04:52584000-52758000 | chr06:60270000-60730000 |
| chr05:00011000-00019000 | chr02:00098000-00161000 |
| chr05:01604000-01644500 | chr02:01743500-01763000 |
| chr05:23827000-23861000 | chr01:71990000-71999000 |
| chr05:63725500-63729500 | chr02:73500500-73530500 |
| chr07:05108500-05221500 | chr19:43107500-43203000 |
| chr07:20826000-20913000 | chr19:09129000-09310500 |
| chr08:60361500-60397500 | chr16:50593500-50623500 |
| chr10:00423000-00450000 | chr07:63525000-63590500 |
| chr10:46372000-46485000 | chr13:16316000-16485000 |
| chr10:61223500-61260500 | chr13:01556000-01605500 |
| chr12:31495000-31558500 | chr11:28565000-28591000 |
| chr12:54372500-54422000 | chr11:07502000-07576000 |
| chr14:50558500-50577500 | chr18:46614000-46673000 |
| chr15:20508000-20634000 | chr17:17172000-17284000 |
| chr20:28045000-28056000 | chr02:30553500-30571000 |

**Supplementary reference**

1. Vandepoele, K., Simillion, C. & Van de Peer, Y. Detecting the undetectable: uncovering duplicated segments in Arabidopsis by comparison with rice. *Trends Genet.* **18,** 606–608 (2002).

**Description of additional supplementary files**

Title: Supplementary Data 1.

Description: Statistics of repeat compositions of the perilla genomes.

Title: Supplementary Data 2.

Description: List of species used in phylogenetic tree and gene family analysis.

Title: Supplementary Data 3.

Description: Cross-validation of 19 inversions by Illumina draft assemblies.

Title: Supplementary Data 4.

Description: List of 29 HEs identified in perilla population.

Title: Supplementary Data 5.

Description: List of 527 genic HEs.

Title: Supplementary Data 6.

Description: List of three de novo non-homeologous exchange events.

Title: Supplementary Data 7.

Description: Summary of the 191 perilla accessions.

Title: Supplementary Data 8.

Description: List of candidate genes involving in TAG biosynthesis.

Title: Supplementary Data 9.

Description: List of candidate genes involved in plant homologous crossover.

Title: Supplementary Data 10.

Description: List of candidate genes for essential oil biosynthesis in perilla.
