# Supplementary material for: Incipient diploidization of the medicinal plant Perilla within 10,000 years
Source: Nat Commun. 2021 Sep 17;12:5508. doi: 10.1038/s41467-021-25681-6 (PMC8448860; doi:10.1038/s41467-021-25681-6)
Supplement: Supplementary file 4 — Description of Additional Supplementary Data [file 41467_2021_25681_MOESM4_ESM.pdf]

Description of Additional Supplementary Files

File Name: Supplementary Data 1

Description: Statistics of repeat compositions of the perilla genomes.

File Name: Supplementary Data 2

Description: List of species used in phylogenetic tree and gene family analysis.

File Name: Supplementary Data 3

Description: Cross-validation of 19 inversions by Illumina draft assemblies.

File Name: Supplementary Data 4

Description: List of 29 HEs identified in perilla population.

File Name: Supplementary Data 5

Description: List of 527 genic HEs.

File Name: Supplementary Data 6

Description: List of three de novo non-homeologous exchange events.

File Name: Supplementary Data 7

Description: Summary of the 191 perilla accessions.

File Name: Supplementary Data 8

Description: List of candidate genes involving in TAG biosynthesis.

File Name: Supplementary Data 9

Description: List of candidate genes involved in plant homologous crossover.

File Name: Supplementary Data 10

Description: List of candidate genes for essential oil biosynthesis in perilla.
