# Supplementary material for: Incipient diploidization of the medicinal plant Perilla within 10,000 years
Source: Nat Commun. 2021 Sep 17;12:5508. doi: 10.1038/s41467-021-25681-6 (PMC8448860; doi:10.1038/s41467-021-25681-6)
Supplement: Supplementary file 5 — Reporting Summary [file 41467_2021_25681_MOESM5_ESM.pdf]

nature research

Corresponding author(s): Yujun Zhang, Shilin Chen

Last updated by author(s): Aug 11, 2021

# Reporting Summary

Nature Research wishes to improve the reproducibility of the work that we publish. This form provides structure for consistency and transparency in reporting. For further information on Nature Research policies, see our Editorial Policies and the Editorial Policy Checklist.

## Statistics

For all statistical analyses, confirm that the following items are present in the figure legend, table legend, main text, or Methods section.

| n/a | Confirmed | |
|---|---|---|
| ☐ | ☒ | The exact sample size (*n*) for each experimental group/condition, given as a discrete number and unit of measurement |
| ☐ | ☒ | A statement on whether measurements were taken from distinct samples or whether the same sample was measured repeatedly |
| ☐ | ☒ | The statistical test(s) used AND whether they are one- or two-sided<br>*Only common tests should be described solely by name; describe more complex techniques in the Methods section.* |
| ☒ | ☐ | A description of all covariates tested |
| ☐ | ☒ | A description of any assumptions or corrections, such as tests of normality and adjustment for multiple comparisons |
| ☐ | ☒ | A full description of the statistical parameters including central tendency (e.g. means) or other basic estimates (e.g. regression coefficient) AND variation (e.g. standard deviation) or associated estimates of uncertainty (e.g. confidence intervals) |
| ☐ | ☒ | For null hypothesis testing, the test statistic (e.g. *F*, *t*, *r*) with confidence intervals, effect sizes, degrees of freedom and *P* value noted<br>*Give P values as exact values whenever suitable.* |
| ☒ | ☐ | For Bayesian analysis, information on the choice of priors and Markov chain Monte Carlo settings |
| ☒ | ☐ | For hierarchical and complex designs, identification of the appropriate level for tests and full reporting of outcomes |
| ☒ | ☐ | Estimates of effect sizes (e.g. Cohen's *d*, Pearson's *r*), indicating how they were calculated |

*Our web collection on statistics for biologists contains articles on many of the points above.*

## Software and code

Policy information about availability of computer code

| | |
|---|---|
| Data collection | No commercial/custom code was used for data collection. |
| Data analysis | Detailed description for softwares used in analysis have been provided in Methods section. Softwares and databases used include: Fermi, Phusion2, SOAPdenovo, SSPACE, Scaff10X, Canu v1.5, Pilon v1.20, Bowtie2, HiC-Pro v2.7.8, BLAST v2.2.28+, MUMmer v3.0, BUSCO v3.02, BWA v0.7.10-r789, Picard v1.119, SAMtools v1.1, GATK v3.5-0-g36282e4, Tandem Repeats Finder v4.07b, Repbase v21.04, RepeatMasker v4-0-6, RepeatProteinMask v4-0-6, RepeatModeler v1-0-8, LTR_retriever, Augustus v3.0.3, GenScan v1.0, Glimmer v3.02, GeneWise v2.2.0, EVidenceModeler v1.1.1, Swiss-Prot v20160809, TrEMBL Release 2016_08, tRNAscan-SE v1.3.1, INFERNAL v1.1rc2, Rfam v12.1, Mercator, MAFFT v7.271, GMAP version 2016-04-04, OrthoMCL v2.0.8, MrBayes v3.2.1, MEGA-CC v7.00-2, DAGchainer r02-06-2008, Circos, MUSCLE v.3.8.31, Codeml, PAML v4.8, ANNOVAR ver.20111120, SnpEff v2.0.5, PHYLIP v3.5c, EIGENSOFT v6.0.1, STRUCTURE v2.3.4, Haploview v4.2, VCFtools v0.1.15, Tophat v2.0.8, RSEM v1.3.2, FaST-LMM. |

For manuscripts utilizing custom algorithms or software that are central to the research but not yet described in published literature, software must be made available to editors and reviewers. We strongly encourage code deposition in a community repository (e.g. GitHub). See the Nature Research guidelines for submitting code & software for further information.

## Data

Policy information about availability of data

All manuscripts must include a data availability statement. This statement should provide the following information, where applicable:

- Accession codes, unique identifiers, or web links for publicly available datasets
- A list of figures that have associated raw data
- A description of any restrictions on data availability

This Whole Genome Shotgun projects have been deposited at DDBJ/ENA/GenBank under the accessions QFCC00000000 (PF40), QIYW00000000 (PC02), and SDAM00000000 (PC99).

# Field-specific reporting

Please select the one below that is the best fit for your research. If you are not sure, read the appropriate sections before making your selection.

☒ Life sciences ☐ Behavioural & social sciences ☐ Ecological, evolutionary & environmental sciences

For a reference copy of the document with all sections, see nature.com/documents/nr-reporting-summary-flat.pdf

# Life sciences study design

All studies must disclose on these points even when the disclosure is negative.

| | |
|---|---|
| Sample size | One plant sample was used for genome sequencing of PF40, PC02, and PC99, respectively. For population re-sequencing, we sampled 191 tetraploid accessions representing all perilla taxonomical groups from China. We also collected 7 diploid lines. |
| Data exclusions | For genome assembly, sequences of low-quality were excluded. |
| Replication | For RNA-seq, three biological replicates were used for each sample, i.e. leaves and flowers of the three reference lines PF40, PC02, and PC99. |
| Randomization | No random sampling is required for genome sequencing, because the genome differences are very small within the population, thus any plant is acceptable for genome sequencing. |
| Blinding | Blinding is not applicable in our study because it does not involve subjects which receive different treatments. All experiments were done by analyzing data derived from different biological replicates directly. |

# Reporting for specific materials, systems and methods

We require information from authors about some types of materials, experimental systems and methods used in many studies. Here, indicate whether each material, system or method listed is relevant to your study. If you are not sure if a list item applies to your research, read the appropriate section before selecting a response.

### Materials & experimental systems

| n/a | Involved in the study | |
|---|---|---|
| ☒ | ☐ | Antibodies |
| ☒ | ☐ | Eukaryotic cell lines |
| ☒ | ☐ | Palaeontology and archaeology |
| ☒ | ☐ | Animals and other organisms |
| ☒ | ☐ | Human research participants |
| ☒ | ☐ | Clinical data |
| ☒ | ☐ | Dual use research of concern |

### Methods

| n/a | Involved in the study | |
|---|---|---|
| ☒ | ☐ | ChIP-seq |
| ☐ | ☒ | Flow cytometry |
| ☒ | ☐ | MRI-based neuroimaging |

## Flow Cytometry

### Plots

Confirm that:

☒ The axis labels state the marker and fluorochrome used (e.g. CD4-FITC).

☒ The axis scales are clearly visible. Include numbers along axes only for bottom left plot of group (a 'group' is an analysis of identical markers).

☒ All plots are contour plots with outliers or pseudocolor plots.

☒ A numerical value for number of cells or percentage (with statistics) is provided.

## Methodology

| | |
|---|---|
| Sample preparation | Nuclei were isolated from young leaves and stained by propidium iodide for 15 minutes. |
| Instrument | CyFlow Cube 6 |
| Software | CyFlow Cube 6 |
| Cell population abundance | Abundance >10000 cells were collected for a sample. Total nuclei populations were gated using relative fluorescence intensity. |
| Gating strategy | Total nuclei populations were gated using PI intensity. In PI+ single cells, the proportions of nuclei of a sample and its contrast were determined based on their PI intensity. |

☒ Tick this box to confirm that a figure exemplifying the gating strategy is provided in the Supplementary Information.
